# Ranid Herpesvirus 3 Infection in Common Frog *Rana temporaria* Tadpoles

**DOI:** 10.3201/eid2906.230255

**Published:** 2023-06

**Authors:** Francesco C. Origgi, Annette Taugbøl

**Affiliations:** University of Messina, Messina, Italy (F.C. Origgi);; University of Bern, Bern, Switzerland (F.C. Origgi);; Norwegian Institute for Nature Research, Lillehammer, Norway (A. Taugbøl)

**Keywords:** ranid herpesvirus 3, common frog, tadpole, amphibian, disease, wildlife, viruses, zoonoses, Norway

## Abstract

Ranid herpesvirus 3 (RaHV3) is a recently discovered virus associated with skin disease in frogs. We detected RaHV3 DNA in free-ranging common frog (*Rana temporaria*) tadpoles, consistent with premetamorphic infection. Our finding reveals a critical aspect of RaHV3 pathogenesis, relevant for amphibian ecology and conservation and, potentially, for human health.

Infectious diseases have been identified as relevant stressors contributing to the ongoing global amphibian decline (*1*). The collapse of amphibian communities translates into a dramatic loss of biodiversity and critical biomass, which eventually could affect human health (*2*). Ranaviruses and chytrid fungi are primary amphibian pathogens that are causing extinction or extirpation of local amphibian populations worldwide (*3*,*4*). It is likely that other infectious organisms, yet to be characterized, might play a similar role.

Recently, 2 novel alloherpesviruses have been discovered: ranid herpesvirus 3 (RaHV3, *Batravirus* *ranidallo3*) in the common frog (*Rana temporaria*) and bufonid herpesvirus 1 in the common toad (*Bufo bufo*). Both viruses are associated with proliferative skin disease (*5*–*7*). RaHV3 is invariably associated with gray patchy skin proliferations corresponding to areas of epidermal hyperplasia (Figure 1) (*7*). Lesions vary in severity and size, but their clinical significance in adult frogs is unknown. Equally unclear is the potential effect of the lesions on overall host fitness, reproductive success, and susceptibility to other infectious agents.

**Figure 1:**
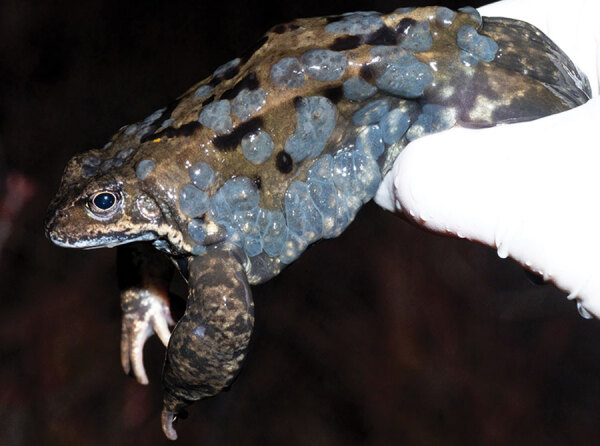
Ranid herpesvirus 3 infection in common frog *Rana temporaria* tadpoles, Norway. Image shows a large number of multifocal to coalescent, mildly elevated, gray patches (epidermal hyperplasia) extending over most of the integument, particularly clustering along the left flank. Between gray areas is normally pigmented skin. Image copyright © Jeroen van der Kooij.

Pathogenesis of RaHV3 is only partially understood (*7*); the actual route and timing of infection is unknown. In a transmission study performed on postmetamorphic common frogs, no specific lesions could be observed after RaHV3 inoculation (F.C. Origgi, unpub. data). Furthermore, experimental transmission studies of ranid herpesvirus 1 (RaHV1, *Batravirus* *ranidallo1*), the first characterized amphibian herpesvirus (*8*), indicated that the virus-associated renal adenocarcinoma most likely occurred when amphibians were infected during the early embryonic stage (premetamorphosis), but not in adult or juvenile stages (postmetamorphosis) (as reviewed in *9*). We investigated the potential occurrence of RaHV3 infection in premetamorphic free-ranging common frogs.

## The Study

**Figure 2:**
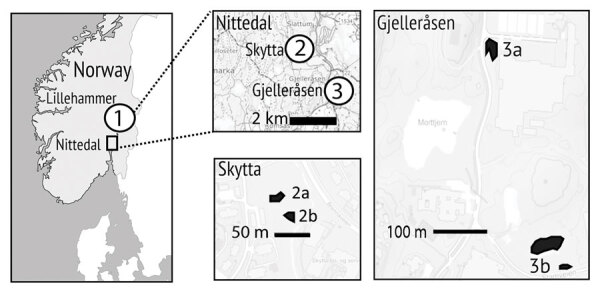
Location of sampling areas in study of ranid herpesvirus 3 infection in common frog *Rana temporaria* tadpoles, Norway. We collected 14 sample batches of free-ranging tadpoles (3–13 tadpoles per batch) either directly from or in close (<10 m) proximity to 5 ponds in Norway in 2022, where adult frogs with lesions consistent with ranid herpesvirus 3 (RaHV3) infection were observed earlier in the year. Maps show locations of ponds in Lillehammer, Skytta, and Gjelleråsen and the distances (in meters) between them. The 2 ponds marked collectively as 3b are linked by marshland in which adult frogs breed independently and are, therefore, treated as 1 complex. We found 2 of 14 sampled tadpole batches were positive for RaHV3 by using PCR, corresponding to pond areas in Lillehammer and Skytta. After testing each tadpole in the positive batches, we identified 6 of 13 tadpoles from the Lillehammer pond and 1 of 4 from the Skytta pond that were RaHV3-positive.

We collected 14 sample batches of free-ranging tadpoles (3–13 tadpoles per batch), either directly from or in close (<10 m) proximity to 5 ponds in Norway in 2022, where adult frogs with lesions consistent with RaHV3 infection were observed earlier in the year (Figure 2) (F.C. Origgi, unpub. data). We sampled the ponds 2–3 times during early June through mid-July 2022 (Appendix Table). The Gosner developmental stages for tadpoles ranged from stages 26–36.

We collected and humanely euthanized tadpoles by using tricaine methanesulfonate in strict accordance with the Animal Welfare Act (§4 in FOR-2003-03-14-349) of Norway and then preserved them in 96% ethanol. In the laboratory, we bisected each tadpole with a scalpel; we extracted DNA from 1 half as previously described (*5*) and fixed the other half in 10% buffered formalin. We amplified the partial RaHV3 genome sequence as previously described (*5*) and by using a new quantitative PCR protocol (Appendix). Initially, we extracted DNA from 3–5 tadpoles from each sampling date and pond location; if we obtained a positive signal for RaHV3 by PCR, we tested all tadpoles collected from the same location. We processed the fixed-tissues, embedded them in paraffin, prepared 5 µm–thick sections, and stained the sections with hematoxylin and eosin in accordance with the standard protocol used at the Vetsuisse Faculty, University of Bern.

After qualitative and quantitative PCR on tadpole DNA (n = 77 samples), we found 2 of 14 sampled batches were positive for RaHV3, corresponding to 2 of the 5 tested pond locations (Lillehammer and Skytta) (Figure 2). After testing each tadpole in the positive batches, we identified 6 of 13 tadpoles from the Lillehammer pond and 1 of 4 from the Skytta pond that were positive for RaHV3 DNA; genome equivalents ranged from 2 × 10^1^ to 2 × 10^7^. After sequencing amplicons obtained by using qualitative PCR (*5*), we found a 100% match with the reference strain RaHV3_FO1_2015 (Genbank accession no. NC_034618.1).

We did not observe differences in histological sections of RaHV3 PCR-positive and PCR-negative tadpoles. However, RaHV3-associated changes might have been masked by the advanced autolysis observed in the examined tissue sections.

## Conclusions

Finding genomic RaHV3 DNA in free-ranging tadpoles is a major step toward understanding the pathogenesis of RaHV3-associated disease. In particular, this result supports the hypothesis that infection occurs during the frog’s premetamorphic embryonic or larval stages. Experimental RaHV1 infection of leopard frogs (*Lithobates pipiens*) was successful only during the early embryonic stages (*9*). Why premetamorphic frogs are presumptively more susceptible to herpesvirus infections is unclear. The lack of keratinized skin in tadpoles (*10*) and the substantial immune system differences between premetamorphic and postmetamorphic life stages might partially explain the susceptibility of premetamorphic frogs to natural infection (*11*). Studies performed with RaHV1 did not clarify the natural route of infection because the embryos were experimentally inoculated with the virus in the pronephros, which is unlikely to mimic what occurs in nature (*9*). RaHV3-infected adult frogs are known to release a large number of virions embedded in sloughed keratinocytes (*5*,*7*), which could eventually be ingested by tadpoles. However, the possibility that oral ingestion of RaHV3 would cause tadpole infection, similarly to what has been shown for Ranavirus, a major amphibian pathogen (*12*), will need to be ruled out by a transmission study.

All 5 tadpole populations were collected in or near ponds where infected adults had been confirmed earlier in the Spring, but only 2 tadpole populations were RaHV3-positive and only at 1 timepoint for each pond (2 of 14 samples in total). Among the positive samples, the Lillehammer population had ≈50% and the Skytta population had ≈25% positivity rates. Reasons for discrepancies in incidence of RaHV3 infection between the different sampled populations remain unclear. Our finding suggests either sporadic RaHV3 infection within different tadpole populations, high virus lethality in infected tadpoles, or both. According to the second hypothesis, RaHV3-positive tadpoles would be difficult to detect in field conditions, because they would rapidly die and be scavenged. Herpesviruses, including alloherpesviruses, infecting a variety of animal species generally cause more severe disease and death in young, immature individuals than in adult hosts, (*13*). Accordingly, RaHV3 might be fatal to a large proportion of infected tadpoles resulting in low detection rates in the remaining viable tadpole population. No obvious tadpole die-offs had been recently reported at the sampling sites; however, those sites are not presently monitored.

In conclusion, our study opens a new venue for understanding RaHV3 pathogenesis and potential effects of RaHV3 infections on premetamorphic and postmetamorphic life stages of amphibian hosts. Understanding the short- and long-term consequences of RaHV3 and other herpesvirus infections on frog populations will be critical for amphibian conservation programs, maintaining biodiversity, and, in turn, supporting human and planetary health (*2*).

Appendix Additional information for ranid herpesvirus 3 infection in common frog *Rana temporaria* tadpoles, Norway.
