# Supplementary material for: Ranid Herpesvirus 3 Infection in Common Frog Rana temporaria Tadpoles
Source: Emerg Infect Dis. 2023 Jun;29(6):1228–31. doi: 10.3201/eid2906.230255 (PMC10202858; doi:10.3201/eid2906.230255)
Supplement: Appendix — Additional information for ranid herpesvirus 3 infection in common frog Rana temporaria tadpoles, Norway. [file 23-0255-Techapp-s1.pdf]

Article DOI: https://doi.org/10.3201/eid2906.230255

*EID cannot ensure accessibility for supplementary materials supplied by authors. Readers who have difficulty accessing supplementary content should contact the authors for assistance.*

# Ranid Herpesvirus 3 Infection in Common Frog *Rana temporaria* Tadpoles, Norway

## Appendix

## Additional Methods

### Quantitative PCR Primer and Probe Design

A forward (FW-5′-GCAGGACACAATTCAAGCGG-3′) and reverse (RV-5′-TTTACTGCGGTATAGCGCCC-3′) primer set and minor groove binder probe (5′-FAM-TGGCGAACAGTGTCAACAGT-3′) were designed in silico by using Geneious 10.0 software (https://www.geneious.com). We used the DNA polymerase gene of ranid herpesvirus 3 (RaHV3, *Batravirus ranidallo3*) (*1*), a highly conserved gene among the order Herpesvirales, as the DNA template. The amplified amplicon was predicted to be 105 nt.

### Positive PCR Control Synthesis

The positive control consisting of DNA amplified by the selected primers was obtained by qualitative PCR. In brief, the master mix contained 3.75 µL 25 mM $MgCl_2$ solution, 3 µL 10× buffer, 0.4 µL 10 mM dNTP mix, 0.2 µL Firepol DNA polymerase (Solis BioDyne, https://www.solisbiodyne.com), 100 ng of DNA (total DNA from an RaHV3 naturally infected frog) (*1*), and double distilled $H_20$ ($ddH_20$) for a total of 30 µL. PCR included a denaturing step at 94°C for 3 min; then 40 cycles comprising 30 s at 94°C (denaturing step), 30 s at 55°C (annealing step), and 30 s at 72°C (extension step). A final 10 min extension step at 72°C was used to exhaust the polymerase. The samples were then resolved in a 2% agarose gel and examined under ultraviolet light.

The PCR amplicon was then cloned into the pGEM-T-Easy vector (Promega, https://www.promega.com) by following the manufacturer’s instructions. Positive white colonies (blue/white selection) were screened, amplified, and the plasmid was purified by using an

established protocol (*2*). The identity of the amplicon was confirmed by automated Sanger sequencing (both strands) (Microsynth AG, https://www.microsynth.com). The sequenced amplicon matched 100% with the expected amplified partial sequence of the RaHV3 DNA polymerase gene and was 105 nt, as expected.

The molecular weight of the positive control DNA amplicon cloned into the expression vector and the corresponding number of molecules contained in 1 ng DNA were determined by using the following formula (https://bitesizebio.com/20669/how-to-calculate-the-number-of-molecules-in-any-piece-of-dna):

number of copies = ng DNA $\times$ ($6.022 \times 10^{23}$)/length $\times$ ($1 \times 10^{9}$) $\times$ 650.

The amplicon was 105 bp and plasmid was 3015 bp, resulting in $2.97 \times 10^{8}$ plasmid copies per ng plasmid DNA.

#### Real-Time PCR Sensitivity Assay

We prepared 10-fold dilutions of the target plasmid in $ddH_2O$, ranging from $10^6$ to $10^{-2}$ copies/µL, and tested the diluted plasmids by using real-time PCR. Samples were tested in duplicate ($10^6$ to $10^2$ copies/µL), triplicate ($10^1$ copies/µL), or quintuplicate (10° copies/µL). The cycling conditions for real-time PCR were: a denaturation step at 95°C for 3 min, then 45 cycles of a denaturation step at 95°C for 30 s and annealing and extension step at 60°C for 1 min. The reactions were performed on an Applied Biosystems 7500 Fast cycler (Thermo Fisher Scientific, https://www.thermofisher.com). The PCR mix consisted of 10 µL 2× qPCR Master Mix (Thermo Fisher), 900 nmol/L of each primer, 250 nmol/L minor groove binder probe (Thermo Fisher), 100 ng of total DNA (for field samples; 1 µL of plasmid suspension for titration), and $ddH_20$ for a total of 20 µL.

The quantitative PCR protocol enabled the detection of $\geq$1 copy of target plasmid per reaction (cycle threshold 39). The $R^2$ value was 0.99, slope was −3.49, and y intercept was 38.71. The primer efficiency was 93.43% (https://www.thermofisher.com/it/en/home/brands/thermo-scientific/molecular-biology/molecular-biology-learning-center/molecular-biology-resource-library/thermo-scientific-web-tools/qpcr-efficiency-calculator.html).

## References

1. Origgi FC, Schmidt BR, Lohmann P, Otten P, Akdesir E, Gaschen V, et al. Ranid herpesvirus 3 and proliferative dermatitis in free-ranging wild common frogs (*Rana temporaria*). Vet Pathol. 2017;54:686–94. PubMed https://doi.org/10.1177/0300985817705176

2. Origgi FC, Plattet P, Sattler U, Robert N, Casaubon J, Mavrot F, et al. Emergence of canine distemper virus strains with modified molecular signature and enhanced neuronal tropism leading to high mortality in wild carnivores. Vet Pathol. 2012;49:913–29. PubMed https://doi.org/10.1177/0300985812436743


**Appendix Table.** Summary of collection dates, pond locations, and tadpole sample types.

| Sample date | Pond location | Area code | Sample type |
|---|---|---|---|
| 2022 Jun 4* | Lillehammer | 1 | Larvae |
| 2022 Jun 16 | Lillehammer | 1 | Larvae |
| 2022 Jun 4 | Skytta1 | 2a | Larvae |
| 2022 Jun 26 | Skytta1 | 2a | Larvae |
| 2022 Jul 10 | Skytta1 | 2a | Larvae |
| 2022 Jun 4* | Skytta2 | 2b | Larvae |
| 2022 Jun 26 | Skytta2 | 2b | Larvae |
| 2022 Jul 10 | Skytta2 | 2b | Larvae |
| 2022 Jun 4 | Pryddammen | 3a | Larvae |
| 2022 Jun 26 | Pryddammen | 3a | Larvae |
| 2022 Jul 10 | Pryddammen | 3a | Larvae |
| 2022 Jun 4 | Froskedammen | 3b | Larvae |
| 2022 Jun 26 | Froskedammen | 3b | Larvae |
| 2022 Jul 10 | Froskedammen | 3b | Larvae |

*Dates and locations where tadpole sample batches were positive for ranid herpesvirus 3.
